# tRNA regulatory ecosystem states shape tissue and circulating small-RNA landscapes in gastric cancer

**DOI:** 10.3389/fcell.2026.1960697

**Published:** 2026-10-02

**Authors:** Jie Liu, Yangyang Chen, Jiaqing Hu, Xujin Wei, Xiaoling Zheng

**Affiliations:** 1 Digestive Endoscopy Center, Fuzhou University Affiliated Provincial Hospital, Fuzhou, Fujian, China; 2 Endoscopic Center, The First Affiliated Hospital, Fujian Medical University, Fuzhou, Fujian, China; 3 Endoscopic Center, National Regional Medical Center, Binhai Campus of the First Affiliated Hospital, Fujian Medical University, Fuzhou, Fujian, China

**Keywords:** gastric cancer, serum biomarker, small-RNA sequencing, tiRNA, tRNA regulation, tRNA-derived fragments, tumour ecosystem

## Abstract

**Background:**

Gastric cancer is a heterogeneous disease shaped by the co-evolution of malignant epithelial programs, stromal remodeling, and immune contexture. Transfer RNA (tRNA) regulation sits at a strategic junction in this biology: it governs translational capacity and selectivity while generating tRNA-derived fragments (tRFs) and tRNA halves (tiRNAs)—a family of regulatory small RNAs detectable in both tissue and blood.

**Objective:**

We asked whether the coordinated machinery responsible for tRNA transcription, maturation, modification, charging, and cleavage defines biologically meaningful gastric cancer ecosystem states, and whether a corresponding circulating small-RNA phenotype can be detected across independent cohorts of chronic atrophic gastritis (CAG), early gastric cancer (EGC), and gastric cancer (GC).

**Methods:**

We integrated transcriptomic, genomic, proteomic, and tissue small-RNA data across complementary gastric cancer series and localized the program in single-cell expression maps. The clinical arm comprised independent serum samples from CAG, EGC, and GC participants.

**Results:**

A 70-gene tRNA-regulatory program resolved three ecosystem states (TRES-1, TRES-2, and TRES-3). TRES-3 exhibited high tRNA-regulatory activity coupled with proliferative and DNA-repair protein expression, whereas TRES-1 was enriched for receptor-signaling and cell-cycle-restraint proteins. These states also differed in copy-number alterations and non-synonymous variant burden at the program level. Direct tissue small-RNA profiling revealed a marked expansion of tRF output and repertoire breadth in TRES-2 and TRES-3, with state-specific tRF classes, parent-tRNA modules, and exact sequences. A shared set of 446 exact tRF sequences connected the tissue and serum catalogues. In an independent gastric cancer series, the three-state architecture aligned with ACRG molecular subtypes, RNA cargo selection, extracellular vesicle release, and stress-response programs. Single-cell mapping placed tRNA-regulatory activity across epithelial, stromal, and immune compartments. In our serum cohort, the 14-30-nt fraction declined from 47.4% in CAG to 37.8% in EGC and 33.6% in GC, while the 31-40-nt fraction rose from 52.6% to 62.2% and 66.4% (absolute Spearman rho = 0.791; BH-adjusted P = 0.0112; exact-permutation P = 0.0214).

**Conclusion:**

tRNA regulation defines gastric cancer ecosystem states with distinct genomic, proteomic, and tissue small-RNA correlates. In our serum cohort, the reciprocal shift in circulating tRF/tiRNA length composition—a decline in the 14-30-nt fraction and a rise in the 31-40-nt fraction—parallels the transition from CAG to EGC to GC. These findings establish a testable molecular framework for paired tissue-serum validation and position circulating tRF/tiRNA architecture as a candidate readout of gastric cancer ecosystem biology.

## 1 Introduction

Gastric cancer remains one of the most lethal malignancies worldwide, in part because it is rarely a single biological entity (Patel et al., 2026; Lordick et al., 2022; Sundar et al., 2025). Its clinical course emerges from interactions among epithelial genotype, differentiation state, stromal composition, immune activity, and therapeutic pressure. Molecular classifications have provided an essential first map of this complexity, distinguishing chromosomal-instability, Epstein-Barr-virus, microsatellite-instability, and genomically stable tumors, alongside the ACRG MSI, mesenchymal (EMT), MSS/TP53-positive, and MSS/TP53-negative groups (Cancer Genome Atlas Research Network, 2014; Cristescu et al., 2015). Yet each classification captures only one dimension of a disease whose histological appearance, invasive behaviour and treatment sensitivity arise from the coordinated behaviour of many cell populations.Immunotherapy combined with chemotherapy has improved outcomes in advanced gastric cancer (Rha et al., 2023).

Recent single-cell and spatial atlases have made this point especially clear. Gastric tumours contain epithelial lineages with different differentiation trajectories, fibroblast populations with distinct extracellular-matrix programmes, vascular niches, myeloid states and lymphoid populations that are organised in a tumour-specific manner (Kumar et al., 2022; Huang et al., 2023; Li et al., 2022; Wang et al., 2021; Liu et al., 2024). These studies have changed the question from whether gastric cancer is heterogeneous to how disparate molecular processes are integrated within a living tumour ecosystem. The answer is likely to involve regulatory systems that operate upstream of individual messenger RNAs and can coordinate large functional outputs across multiple compartments.

tRNA biology offers such a system. Beyond their classical role as adaptors in translation, tRNAs are dynamically regulated through transcription, end processing, base modification, aminoacylation, subcellular trafficking and stress-responsive cleavage (Suzuki, 2021; Linder et al., 2025; Ramirez-Moya et al., 2025). These steps influence codon decoding, translational speed, proteome composition and the handling of cellular stress. In cancer, altered tRNA modification or anticodon editing can support codon-biased translation of growth-promoting transcripts, reconfigure proteostasis and permit cells to adapt to nutrient, oxidative and genotoxic stress (Linder et al., 2025; Ramirez-Moya et al., 2025; Wang et al., 2026). A programme built around the tRNA machinery therefore has the potential to reveal an integrated biological state rather than a collection of isolated RNA-processing genes.

The small-RNA products of tRNA metabolism make this question particularly attractive in gastric cancer. tRFs and tiRNAs are generated with characteristic cleavage positions and lengths, can regulate translation and RNA stability, and participate in stress adaptation, proliferation, invasion and therapeutic response (Yu et al., 2021; Gong et al., 2023; Chen et al., 2024). Several gastric-cancer studies have identified individual tRFs with cell-biological functions, including tRF-Val and tRF-Tyr, while plasma assays have shown that a sequence-defined tRF can be quantified across the continuum from non-neoplastic gastric disease to cancer (Kohansal et al., 2022; Zhang et al., 2023; Cui et al., 2022; Cui et al., 2025). These observations create an important opportunity: a tissue-centred framework for tRNA regulation could be linked to a serum readout that is accessible in routine clinical material.

The field nevertheless lacks an integrated account of how tRNA-regulatory machinery, tissue tRF output and circulating tRF/tiRNA architecture relate to one another in gastric cancer. Most reports focus on one candidate fragment, one enzyme or one molecular layer. Such studies are valuable, but they do not reveal whether the tRNA system is organised as a broader ecosystem programme, whether its small-RNA products differ across tumour states, or where this programme resides within the multicellular tumour landscape. Addressing these questions requires a design that moves from regulatory machinery to measured RNA products and finally to clinically collected serum.

Here, we define tRNA-regulatory ecosystem states in gastric cancer using a biologically curated program spanning tRNA transcription, processing, modification, aminoacylation, and fragmentation response. We then characterize the genomic and proteomic features of these states, directly quantify their tissue tRF architecture, examine their relationship to established gastric cancer subtypes, and localize the program across tumor, stromal, and immune lineages. Finally, we connect this tissue framework to serum small-RNA sequencing data from our own CAG-EGC-GC clinical series. This integrated framework thus positions tRNA regulation as a measurable component of gastric cancer ecosystem biology, enabling simultaneous examination of its tissue and circulating manifestations.

## 2 Materials and methods

### 2.1 Study design and molecular cohorts

This study was designed as a layered investigation of tRNA regulation in gastric cancer. The discovery layer used TCGA-STAD to define states from a locked 70-gene tRNA-regulatory programme. Matched molecular profiles were then used to characterise copy-number alteration, somatic mutation burden and protein abundance. Tissue small-RNA output was examined in patient-linked TCGA MINTmap libraries, an exhaustive tRF resource with MINTbase sequence identifiers and parent-tRNA annotations (Loher et al., 2017; Pliatsika et al., 2018). A separate gastric-cancer expression series was used to examine the generality of the state architecture and its relationship with ACRG classes. Single-cell matrices from 40 gastric-cancer specimens were used to map the programme across broad cellular lineages. The clinical-material layer consisted of serum small-RNA sequencing from nine individuals representing CAG, EGC and GC.

### 2.2 Definition of the tRNA-regulatory programme and ecosystem states

The 70-gene programme was specified before state learning and represented five connected dimensions of tRNA biology: RNA polymerase III transcription, precursor processing, nucleotide modification, aminoacylation and fragmentation/stress response. TCGA-STAD tumour expression values were standardised gene-wise. We used consensus k-means with 200 resampling iterations across candidate solutions from K = 2 to K = 5, resampling 80% of tumours and 80% of programme genes at each iteration. State selection integrated consensus stability, the proportion of ambiguous clustering, silhouette structure and the requirement that every state contained a substantial tumour population.

The selected three-state solution was labelled TRES-1 to TRES-3 according to its programme-centroid trajectory. Labels were fixed before comparison with clinical, molecular, protein, tissue small-RNA or serum variables. The random seed was 20260806. Complete programme membership, module labels, state assignments, centroids and K = 2-5 stability diagnostics are provided in Supplementary Tables S2-S4.

### 2.3 Genomic and protein characterisation

Programme-level GISTIC copy-number calls, non-synonymous exome variant counts and reverse-phase protein array (RPPA) abundance measurements were linked to the fixed state assignments by patient identifier. Copy-number analysis focused on the locked programme genes and summarised the fraction altered in each tumour. Protein analysis assessed the 206 RPPA targets available in the overlapping cases and used Benjamini–Hochberg correction across the complete target family. State contrasts were evaluated by Kruskal–Wallis testing, with effect sizes retained alongside P values to describe the magnitude and direction of separation.

### 2.4 Tissue tRF architecture and tissue-serum sequence catalogue

MINTmap exclusive-tRF expression tables were linked to the TRES assignment. One primary-tumour library was retained per patient by original input-read depth, producing a patient-level tissue small-RNA cohort of 409 representatives. We examined total tRF abundance, the number of detected fragments, tRF-class composition, parent-tRNA source families and exact MINTbase fragments. The pre-specified testing family comprised two aggregate-output endpoints, three tRF-class endpoints, two source-origin endpoints, 48 source-tRNA families and 805 filtered exact fragments; all P values were adjusted across this full inventory.

Exact sequences in the tissue catalogue were compared with the serum sequence-coordinate catalogue. The shared repertoire was defined by identical nucleotide strings and parent-tRNA annotation rather than by a broad family label.

### 2.5 State architecture in independent tumours and single-cell maps

External projection used GSE62254 (Affymetrix Human Genome U133 Plus 2.0, GPL570; n = 300). Expression was taken from the GEO series matrix, mapped to genes with the platform annotation, and represented by the maximum-IQR probe per gene. The frozen TCGA centroids were applied without refitting to 69/70 represented genes; gene-wise standardisation within GSE62254 preceded Pearson correlation to each centroid, and the largest correlation defined assignment. Assignment correlations are reported as confidence information. For single-cell localisation, processed matrices from GSE183904 (40 supplied samples; 158,641 cells) were summarised using fixed broad-lineage marker panels and sample-by-lineage medians, requiring at least 20 called cells per stratum. This descriptive analysis locates programme expression across epithelial/tumour, CAF/ECM, endothelial and immune compartments. GSE206785 (24 tumour/normal pairs; 20 eligible pairs) was retained as a supplementary paired sensitivity analysis.

### 2.6 Serum small-RNA sequencing and statistical analysis

Independent serum samples were available from CAG1-3, EGC1-3 and GC1-3. All samples were collected and processed under the same prespecified standard operating procedure. Total RNA was isolated from serum and assessed by NanoDrop ND-1000 and agarose-gel electrophoresis as reported by the sequencing provider. Modification-aware pretreatment comprised 3′-aminoacyl deacylation, 3′-cyclic-phosphate removal, 5′-hydroxyl phosphorylation and m1A/m3C demethylation, followed by sequential adapter ligation, reverse transcription, PCR amplification and approximately 134-160-bp size selection. Libraries were assessed with an Agilent 2,100 Bioanalyzer and sequenced as single-end 50-nt reads on an Illumina NextSeq 550. Adapters were trimmed and reads outside 14-40 nt were discarded with cutadapt. Remaining reads were aligned first to mature tRNAs and then precursor tRNAs with Bowtie, allowing one mismatch; unaligned reads were subsequently assessed against miRNA references with miRDeep2. Cytoplasmic tRNAs came from GtRNAdb, mitochondrial tRNAs were predicted with tRNAscan-SE, mature references had introns removed and terminal CCA added, and precursor references included 40-nt genomic flanks. Vendor-reported counts were normalised as CPM. Ordered cross-sectional association across CAG, EGC and GC was evaluated by Spearman correlation, exact permutation inference and leave-one-out summaries. Source and fragment analyses were exploratory.

All tests were two-sided. Benjamini–Hochberg adjustment was applied within the stated test families. Kruskal–Wallis analyses report the statistic, exact or asymptotic P value, adjusted q value and epsilon-squared where available; categorical comparisons report chi-square statistics and sample sizes. Tissue tRF tests used RPM or within-library composition and one deterministically depth-selected library per patient. The unusually large significant-fragment fraction is interpreted in light of correlated fragments, library depth and compositional dependence; aggregate output, class, origin and fragment layers were included in one prespecified 860-test global FDR family.

## 3 Results

### 3.1 A 70-gene tRNA-regulatory program defines three distinct ecosystem states in gastric cancer

We began by asking whether the collective activity of tRNA-related genes—spanning transcription, processing, modification, aminoacylation, and stress-responsive cleavage—could resolve biologically meaningful subgroups within gastric cancer. Using a locked 70-gene program (Figure 1a), we applied consensus clustering to 412 TCGA-STAD tumors without reference to any clinical or outcome data.

**FIGURE 1:**
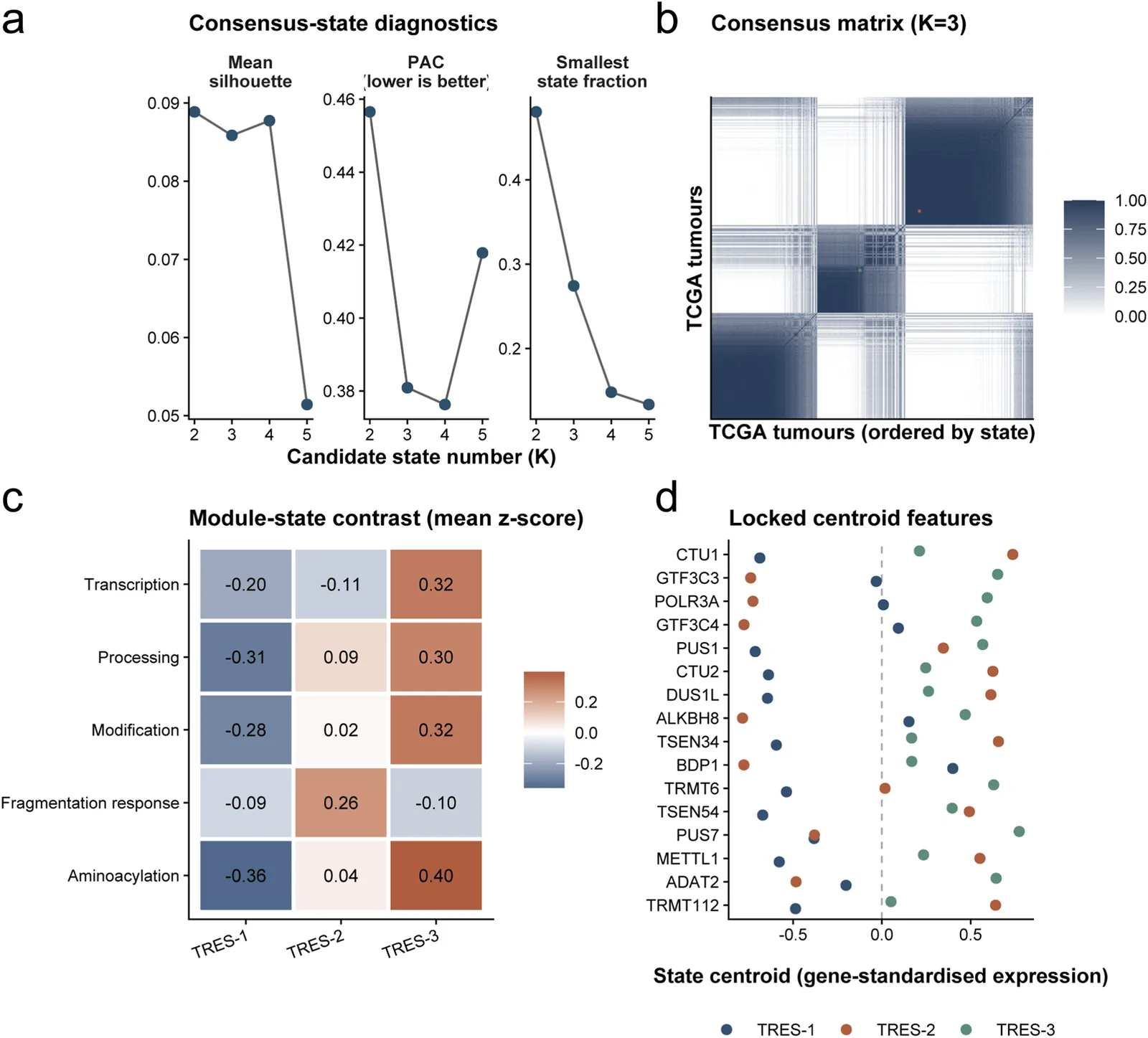
Discovery of tRNA-regulatory ecosystem states in 412 TCGA-STAD tumours. **(a)** Candidate-state diagnostics across K = 2-5, including PAC and mean silhouette; consensus k-means used 200 resamples, 80% of tumours and 80% of genes per iteration, seed 20260806. **(b)** Consensus matrix for K = 3 (TRES-1 n = 163, TRES-2 n = 113, TRES-3 n = 136). **(c)** Mean gene-wise standardised programme/module scores. **(d)** Leading state-centroid genes. State learning was outcome-blind. TRES, tRNA-regulatory ecosystem state.

The analysis converged on a three-state solution. The consensus matrix showed three clearly separated tumor blocks (Figure 1b), and diagnostic metrics across K = 2-5 supported this structure with favorable stability and silhouette values. The largest state, TRES-1, contained 163 tumors; TRES-2 and TRES-3 contained 113 and 136 tumors, respectively. No state was too small to be interpreted as a distinct subgroup.

We next examined what these states represented biologically. Plotting the mean program scores for each state revealed a clear gradient (Figure 1c): TRES-1 occupied the low-activity end, TRES-2 showed an intermediate configuration with enriched processing and fragmentation-response modules, and TRES-3 carried the highest combined tRNA-regulatory activity. This ordering was not driven by a single module but extended across transcription, modification, and cleavage components—suggesting that the states reflect global differences in tRNA system engagement rather than isolated gene-level variation.

To identify the specific genes driving this architecture, we extracted the leading centroid contributors (Figure 1d). CTU1, CTU2, POLR3A, GTF3C3, PUS1, DUS1L, ALKBH8, TSEN34, TSEN54, TRMT6, METTL1, ADAT2, and TRMT112 collectively shaped the TRES trajectory, linking the state axis to tRNA supply (POLR3A), base modification (PUS1, ALKBH8, TRMT6, METTL1), and stress-responsive RNA processing (TSEN34, TSEN54, ADAT2). These genes thus provided an interpretable molecular signature for each state.

Together, these findings established that the tRNA-regulatory program does not vary randomly across gastric tumors. Instead, it resolves into three coherent states that differ in the overall intensity and composition of tRNA-related activity. We next examined whether these states represent genuine molecular subgroups with distinct genomic and proteomic contexts.

### 3.2 TRES states carry distinct genomic and proteomic identities

To test whether the three states are embedded in broader molecular landscapes, we linked the fixed TRES assignments to available DNA and protein measurements from the same TCGA tumors (Figure 2a). Of the 412 tumors, 410 had GISTIC copy-number data, 408 had exome mutation records, and 333 had RPPA protein profiles.

**FIGURE 2:**
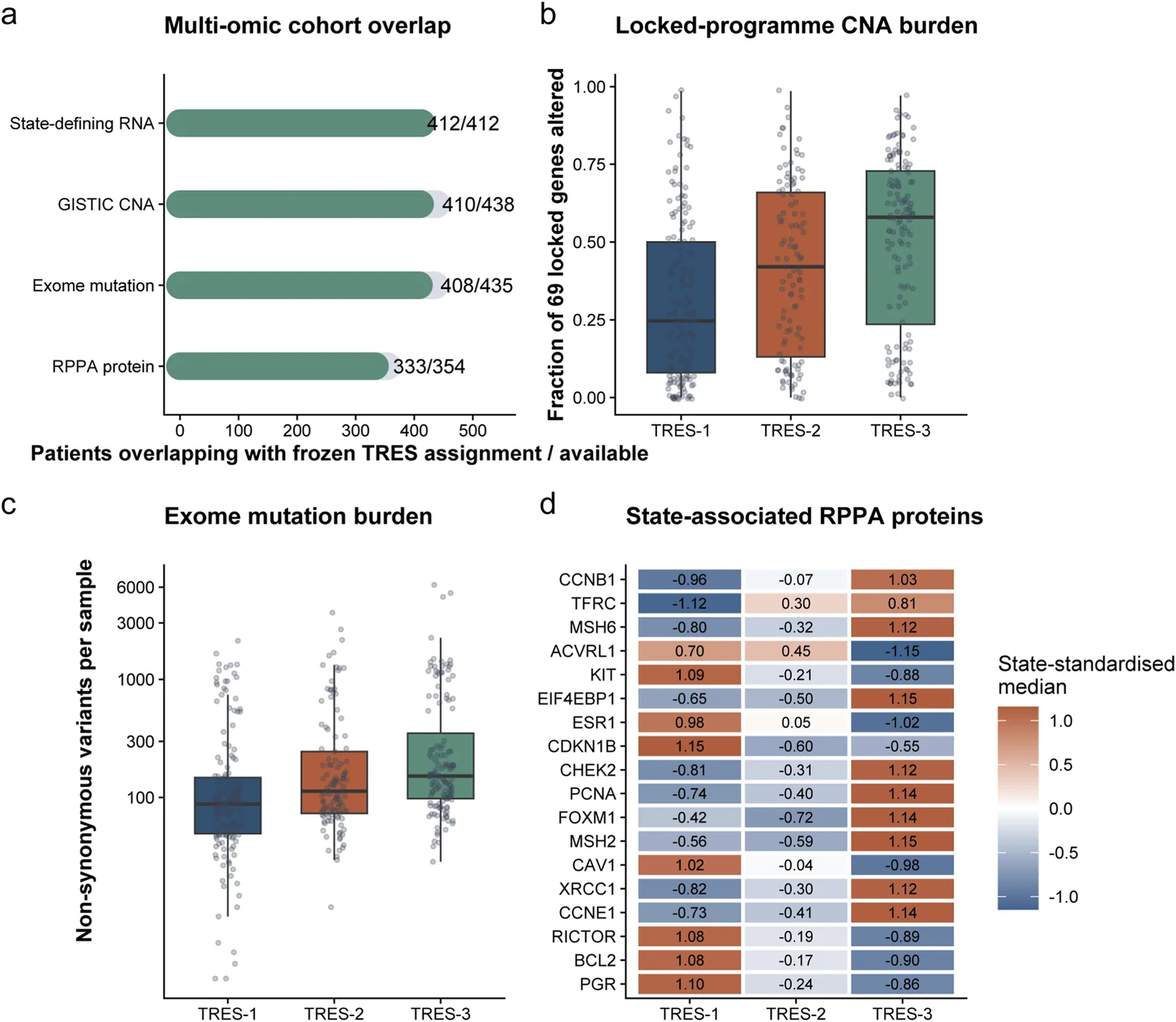
Genomic and proteomic identity of TRES states. **(a)** Overlap of 412 state-assigned tumours with GISTIC (n = 410), mutation (n = 408) and RPPA (n = 333) layers. **(b)** Per-tumour fraction of 69 available locked genes with GISTIC alteration; Kruskal–Wallis P = 1.765x10^-8, global BH q = 5.295x10^-8, epsilon-squared = 0.083. **(c)** Non-synonymous variants per sequenced sample; Kruskal–Wallis P = 2.288x10^-8, epsilon-squared = 0.082. **(d)** State-associated RPPA targets among 206 tested proteins; BH correction was applied across all 206 targets. Missing profiles were excluded analysis-wise.

At the DNA level, we observed that the fraction of locked program genes affected by copy-number alterations differed significantly across states (Figure 2b; Kruskal–Wallis P = 1.765 × 10^−8^, ε^2^ = 0.083). Similarly, the burden of non-synonymous somatic variants varied across the three states (Figure 2c; P = 2.288 × 10^−8^, ε^2^ = 0.082). These results indicated that the TRES axis is not purely an expression-level phenomenon but is grounded in distinguishable genomic configurations.

The protein layer provided functional resolution. TRES-3 tumors showed elevated expression of proteins involved in cell-cycle progression (CCNB1, PCNA, FOXM1), DNA replication and repair (MSH2, MSH6, XRCC1), iron handling (TFRC), and translational control (EIF4EBP1) (Figure 2d). TRES-1, by contrast, was characterized by receptor-signaling proteins (ESR1, PGR) and cell-cycle restraints (CDKN1B, CAV1, BCL2, RICTOR). This reciprocal protein signature suggested a fundamental biological contrast: the high-programme state (TRES-3) aligns with proliferation and genomic maintenance, whereas the low-programme state (TRES-1) reflects a more restrained, signaling-oriented cellular context.

The three TRES states therefore inhabit distinct genomic environments and protein functional spaces. The high-programme state, in particular, sits in a proliferative, replication-competent setting where tRNA supply may become limiting. This molecular coherence justified a more functional investigation: whether the actual output of the tRNA machinery—the tRNA-derived small RNAs—also differs across states.

### 3.3 Tissue tRF abundance and repertoire composition are state-specific

To determine whether mRNA-defined regulatory states are accompanied by measured differences in tRNA-derived RNA products, we examined tissue small-RNA sequencing data from 409 TCGA tumors with available MINTmap profiles. Rather than focusing on a single candidate tRF, we quantified the entire detectable tRF landscape across the three states.

Total tRF output increased sharply from TRES-1 to TRES-2 and TRES-3 (Figure 3a). Median abundance rose from 2,280 RPM in TRES-1 to 4,662 RPM in TRES-2 and 4,323 RPM in TRES-3 (global BH q = 5.16 × 10^−11^). Repertoire breadth followed the same pattern: TRES-1 tumors had a median of 280 detected fragments (RPM ≥1), compared with 391 in TRES-2 and 410 in TRES-3 (q = 3.41 × 10^−9^). The states therefore differed in both total tRF signal and the number of distinct fragments generated.

**FIGURE 3:**
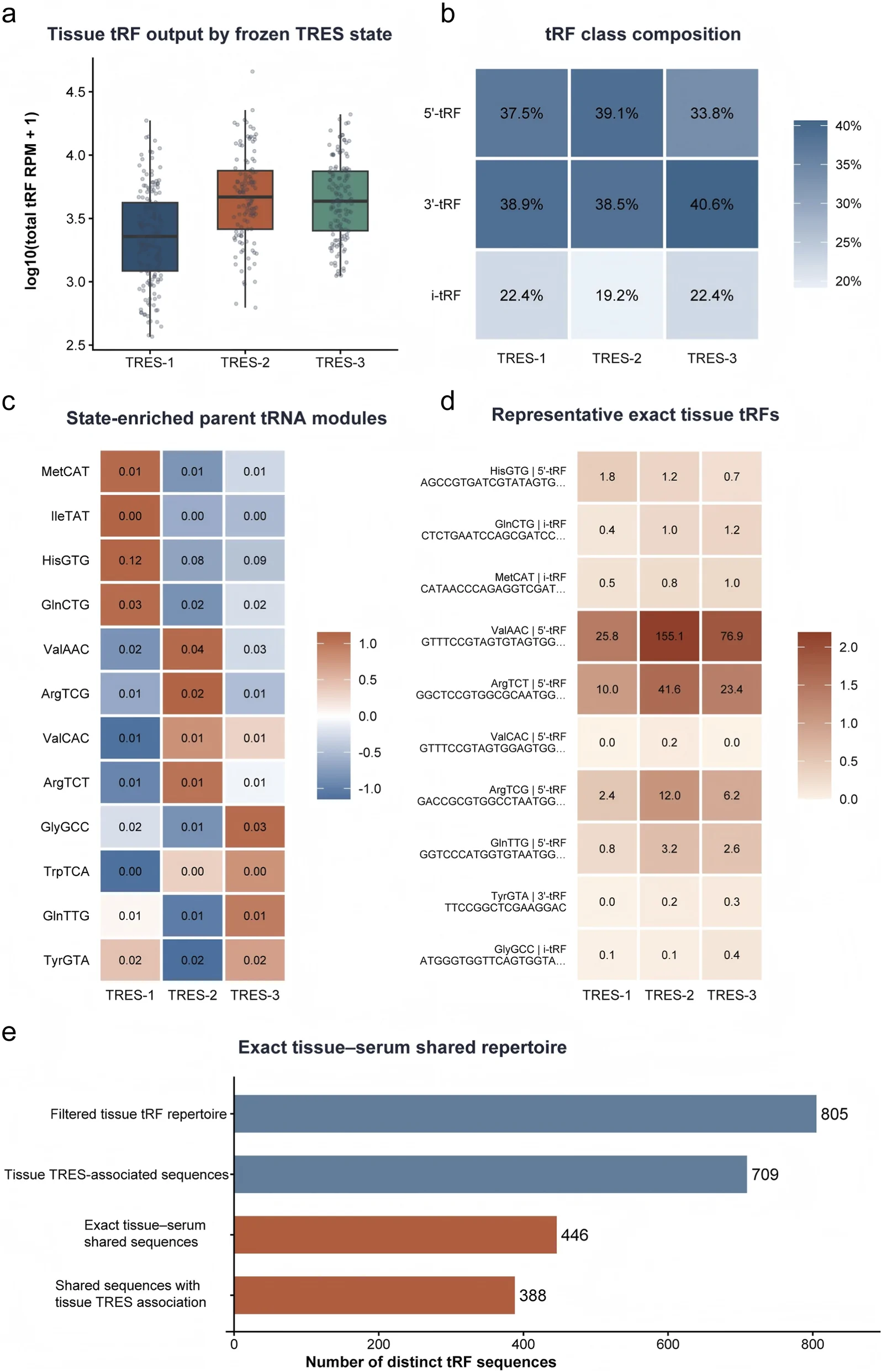
Tissue tRF architecture across TRES states in 409 patient-representative TCGA-STAD MINTmap libraries (TRES-1 n = 162, TRES-2 n = 113, TRES-3 n = 134). **(a)** Total tRF abundance, plotted as log10(total RPM + 1); Kruskal–Wallis tests with epsilon-squared and global BH q values. **(b)** Within-library tRF-class fractions. **(c)** Row-standardised state medians for 12 parent-tRNA source families selected from globally significant source-family tests. **(d)** State-median RPM for representative exact MINTbase sequences; the colour scales in C and D encode different quantities and are not directly comparable. **(e)** Catalogue-level exact-sequence overlap; tissue and serum cohorts were unpaired. All 860 aggregate, class, origin, source and fragment tests formed one prespecified global BH family.

We next examined which classes of tRFs contributed to this difference. Internal tRFs and 5′-tRFs varied significantly across the three states (Figure 3b), indicating that the state signal involves cleavage-site architecture as well as overall output. This places the TRES axis directly within the biology of tRNA processing—the states differ in how tRNAs are cleaved, not only in how much tRNA is expressed.

Mapping the fragments back to their source tRNA families identified twelve families with globally significant state associations (Figure 3c), including Met-CAT, Ile-TAT, His-GTG, Gln-CTG, Val-AAC, Arg-TCG, Val-CAC, Arg-TCT, Gly-GCC, Trp-TCA, Gln-TTG, and Tyr-GTA. Within these families, the representative exact fragments showed distinct patterns of median abundance: His‐GTG was highest in TRES‐1; Val‐AAC, Arg‐TCT, Val‐CAC, Arg‐TCG, and Gln‐TTG were highest in TRES‐2; and Gln‐CTG, Met‐CAT, Tyr‐GTA, and Gly‐GCC were highest in TRES‐3 (Figure 3d). The tissue and serum catalogues shared 446 exact tRF sequences (Figure 3e). These exact sequences make the state architecture experimentally portable for future targeted studies.

The tRNA-regulatory states are thus expressed at the RNA-product level as distinct tRF architectures, bridging gene-expression regulation and actual small-RNA output. To further validate these states as general features of gastric cancer biology, we tested their reproducibility in independent datasets and across different molecular platforms.

### 3.4 The three-state architecture generalizes to independent cohorts and maps to multiple cellular compartments

We projected the frozen TCGA centroids onto 300 tumors from the independent GSE62254 gastric cancer series without refitting the model. The assignment correlated well with the original TCGA templates (Figure 4A), and the resulting groups contained 113, 84, and 103 tumors, respectively—closely mirroring the original proportions.

**FIGURE 4:**
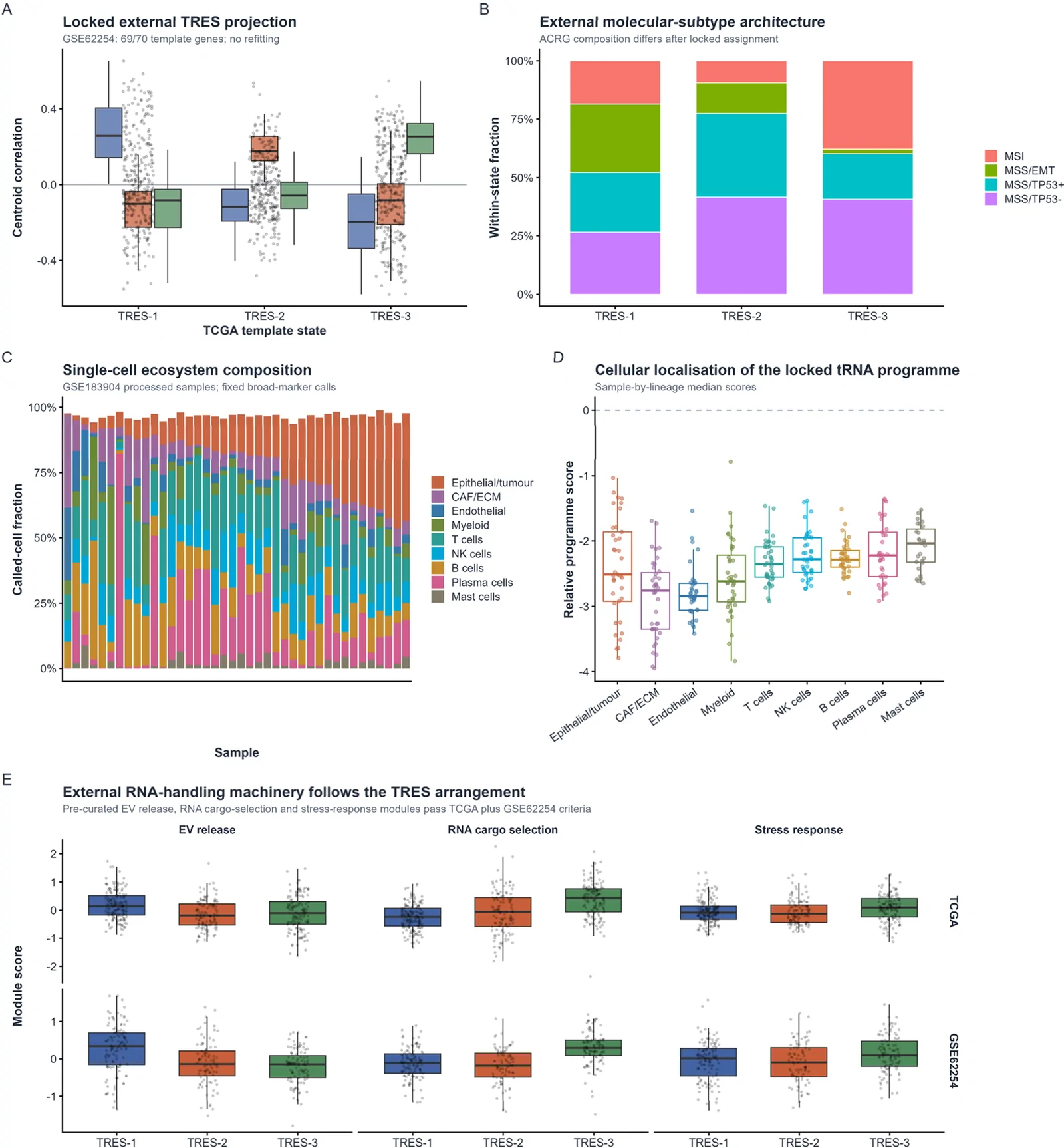
External molecular, cellular and RNA-handling context of TRES states. **(A)** Pearson correlations to frozen TCGA centroids after no-refit projection of 300 GSE62254 GPL570 tumours using 69/70 genes (TRES-1 n = 113, TRES-2 n = 84, TRES-3 n = 103). **(B)** ACRG subtype composition; chi-square P = 1.09x10^-9. **(C)** Broad marker-defined composition across 40 samples (158,641 supplied cells). **(D)** Sample-by-lineage median relative programme scores for strata with ≥ 20 called cells; values locate programme expression and do not imply cell-cell coordination. **(E)** Pre-curated EV-release, RNA-cargo-selection and stress-response module scores in TCGA and GSE62254; Kruskal–Wallis tests and within-cohort BH correction were used, and only modules meeting the cross-cohort criterion are displayed.

The projected states showed a strong relationship with ACRG molecular subtypes (Figure 4B; χ^2^ P = 1.09 × 10^−9^). TRES-1 was enriched for MSS/EMT tumors, TRES-2 was dominated by MSS/TP53-positive and MSS/TP53-negative groups, and TRES-3 was enriched for MSI tumors with minimal EMT representation. This alignment anchored the TRES axis to established gastric cancer taxonomy and confirmed that the state architecture is not unique to TCGA.

We next mapped the locked 70-gene program across major cell lineages using single-cell data from 40 gastric cancer specimens (GSE183904). The program was measurable in epithelial/tumor cells, fibroblasts, endothelial cells, and multiple immune populations (Figures 4C,D). Relative program scores were highest in immune-lineage and epithelial/tumor compartments and lower in endothelial and CAF/ECM compartments. This localization indicates that tRNA regulation is not confined to cancer cells but is distributed across the tumor microenvironment.

We also examined whether the TRES states differ in broader RNA-handling programs—extracellular vesicle release, RNA cargo selection, and stress response. These modules differed across states in both TCGA and GSE62254 under the cross-cohort criterion (Figure 4E). TRES-1 showed higher EV release, whereas TRES-3 showed higher RNA cargo selection and stress response. These patterns place the tRNA-regulatory states within a larger extracellular-RNA biology framework.

Having established a reproducible tissue-level framework that aligns with established subtypes and distributes across multiple cell types, we turned to the translational question of whether a corresponding circulating small-RNA phenotype could be detected in patient serum.

### 3.5 Serum small-RNA sequencing identifies reciprocal length remodelling from CAG to gastric cancer

The clinical arm of this study consisted of serum small-RNA sequencing from nine independent participants—three with chronic atrophic gastritis (CAG), three with early gastric cancer (EGC), and three with gastric cancer (GC). We compared circulating tRF/tiRNA architecture across these three diagnostic groups.

The group-mean 14–40-nt length spectrum revealed a progressive shift in the balance between shorter and longer fragments from CAG to EGC to GC (Figure 5A). Quantitatively, the 14-30-nt fraction declined from 47.4% in CAG to 37.8% in EGC and 33.6% in GC, while the 31-40-nt fraction rose from 52.6% to 62.2% and 66.4% (Figure 5B). Because these two fractions partition the same denominator, they represent a single reciprocal compositional contrast.

**FIGURE 5:**
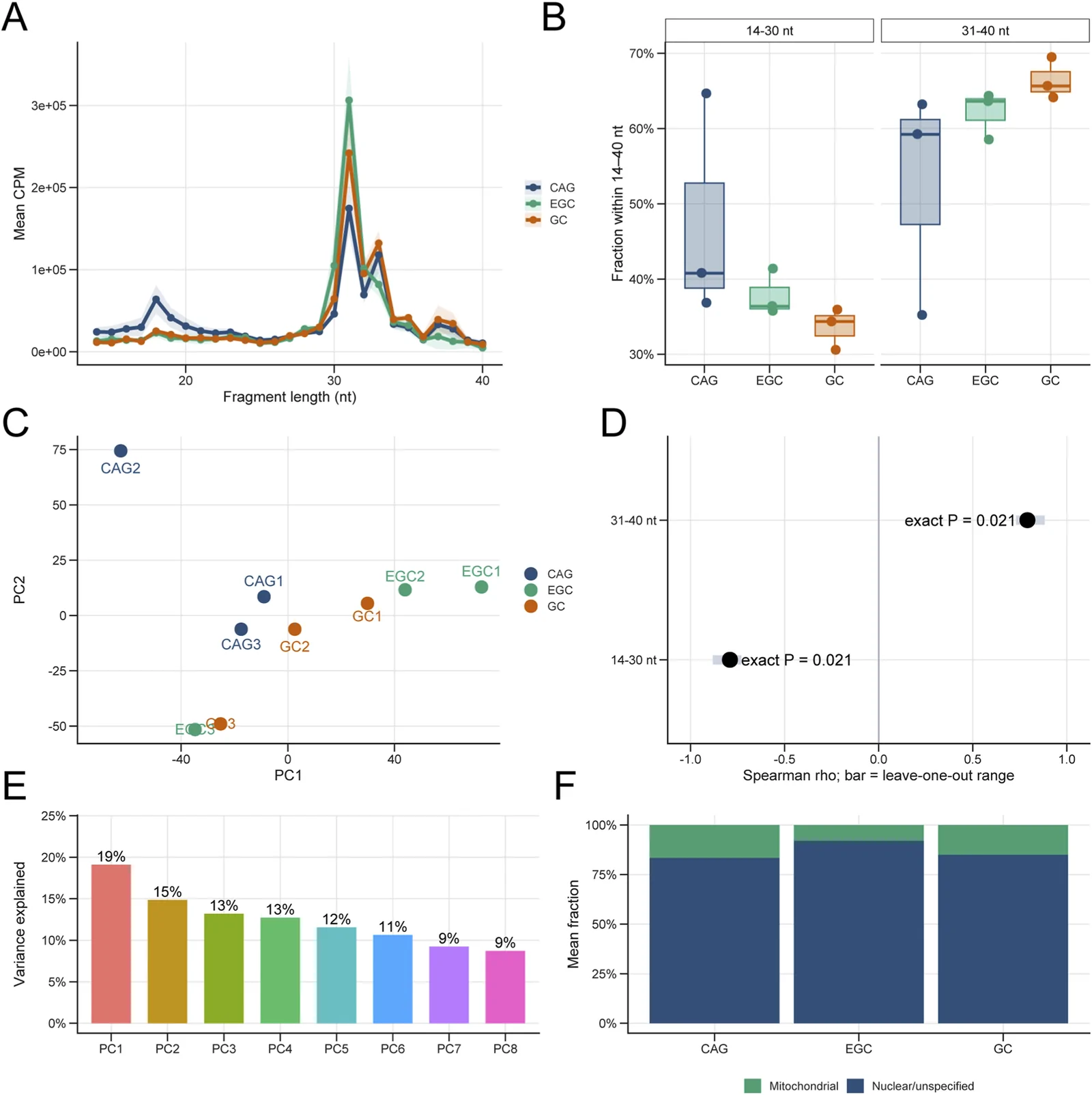
Serum tRF/tiRNA profiling in independent CAG (n = 3), EGC (n = 3) and GC (n = 3) participants. **(A)** Group-mean 14-40-nt CPM spectrum. **(B)** Sample-level fractions within the common 14-40-nt denominator; the 14-30-nt and 31-40-nt fractions are complementary and constitute one reciprocal compositional contrast. **(C)** PCA of log-transformed exact-mapping CPM features. **(D)** Ordered cross-sectional Spearman association (absolute rho = 0.791; BH-adjusted P = 0.0112; exact-permutation P = 0.0214) and leave-one-out ranges. **(E)** Percentage variance explained by PCA components. **(F)** Within-sample mitochondrial and nuclear/unspecified source-origin fractions. Findings are exploratory and do not represent longitudinal progression or diagnostic performance.

Principal component analysis of exact-mapping CPM values provided an orthogonal view of this shift (Figure 5C). Samples separated along the major compositional axis in a manner consistent with the ordered length transition. The cross-sectional association was statistically robust: the reciprocal contrast had absolute Spearman rho = 0.791 (BH-adjusted P = 0.0112; exact-permutation P = 0.0214), and leave-one-out summaries retained the same direction (Figure 5D). The leading PCA components captured a substantial portion of the sequence landscape, confirming that the length shift reflects a major axis of serum variation (Figure 5E). Source-origin analysis further localized the effect: both nuclear/unspecified and mitochondrial-derived tRF/tiRNA fractions contributed to the global shift (Figure 5F).

Circulating tRF/tiRNA architecture thus rebalances across the CAG-EGC-GC sequence. The shift is statistically ordered and visually consistent, although the sample size (n = 3 per group) and the absence of paired tissue samples limit interpretation. We therefore resolved the global length shift into specific source tRNAs and exact fragments to generate sequence-level candidates for future validation.

### 3.6 The serum phenotype resolves to specific source tRNAs and exact sequences

To move from global length composition to sequence-level candidates, we examined individual fragment profiles across the three serum groups. Five exact fragments—Val-AAC, Lys-CTT, Leu-CAG, Leu-CAA, and Arg-ACG—showed reproducible group-patterned CPM differences (Figure 6A), forming a compact panel for future validation.

**FIGURE 6:**
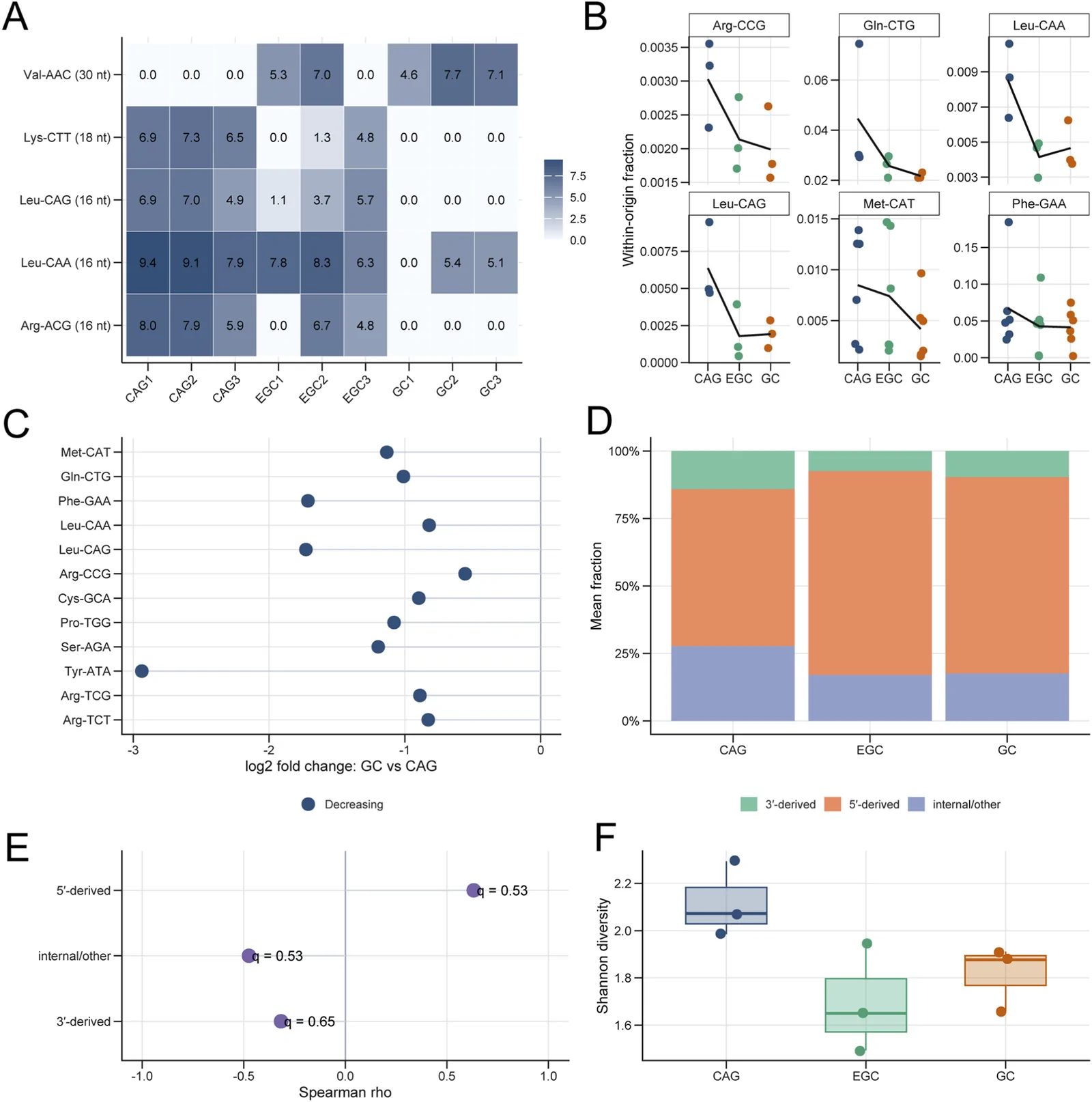
Source and exact-fragment architecture in CAG (n = 3), EGC (n = 3) and GC (n = 3) serum samples. **(A)** Log2(CPM+1) profiles of five sequence-coordinate-audited fragments. **(B)** Group medians of within-sample parent-tRNA source fractions. **(C)** GC-versus-CAG log2 fold changes in source fraction; descriptive effect sizes are shown without diagnostic interpretation. **(D)** Within-sample fragment-architecture fractions. **(E)** Spearman rho for ordered cross-sectional architecture associations with BH adjustment within the displayed family. **(F)** Shannon diversity of amino-acid source fractions. No paired tissue-serum or longitudinal inference is made.

At the parental tRNA level, several source families displayed declining trajectories from CAG to EGC to GC, including Arg-CCG, Gln-CTG, Leu-CAA, Leu-CAG, Met-CAT, and Phe-GAA (Figure 6B). Source-level effect screens highlighted Tyr-ATA, Leu-CAG, and Phe-GAA as among the strongest GC-versus-CAG differentiators (Figure 6C). These families connect the global length shift to interpretable parental origins.

Fragment-structure analysis showed that the serum phenotype involves rebalanced cleavage products across the disease sequence (Figures 6D,E). The architecture categories—including 3′-tRFs, 5′-tRFs, and internal fragments—contributed to the overall trend. Shannon diversity was lower in EGC and GC than in CAG, with the lowest median in EGC (Figure 6F), suggesting that the serum tRF pool becomes more restricted in its source diversity as disease progresses.

These serum findings supply a sequence-resolved candidate map: specific source families, exact fragments, and architectural classes that await orthogonal validation in larger cohorts with paired tissue and blood samples.

## 4 Discussion

This study demonstrates that tRNA regulation organizes gastric cancer biology across multiple molecular layers. A coordinated tRNA-regulatory program resolves three ecosystem states recognizable in genomic, proteomic, and tissue small-RNA data, positions within established gastric cancer subtypes, and links to a serum small-RNA phenotype. The framework connects the machinery of tRNA biology to its RNA products and to a circulating readout.

The three TRES states provide a biologically coherent framework for understanding why tRNA regulation may matter in gastric cancer. TRES-3 combines high program activity with expression of CCNB1, PCNA, FOXM1, MSH2, MSH6, and XRCC1, placing this state in a proliferative, replication-competent, and DNA-repair-rich context. This is precisely the environment in which tRNA transcription, modification, and aminoacylation could become limiting determinants of cell fitness. Highly proliferative cells require sustained and selective translation, while replication stress and metabolic strain increase the demand for an adaptable proteostasis system. Current work on tRNA modifications and anticodon editing supports this view: altered tRNA chemistry can favor decoding of growth-promoting codon programs and remodel the proteome in cancer cells (Suzuki, 2021; Linder et al., 2025; Ramirez-Moya et al., 2025; Wang et al., 2026).

TRES-1 provides the complementary pole of this organization. Its association with ESR1, PGR, CDKN1B, CAV1, BCL2, and RICTOR suggests a cellular context in which receptor signaling, membrane-associated biology, and cell-cycle restraint are more prominent. This contrast is valuable because it prevents the tRNA program from being interpreted as a generic proliferation score. TRES-1 and TRES-3 differ in the balance of regulatory functions that determine how cells meet translational demand, respond to stress, and engage with their microenvironment. TRES-2, with its prominent tissue tRF output, further suggests that high fragment production represents a distinct regulatory configuration rather than a simple midpoint between the two extremes.

Direct tissue tRF profiling is the key element that turns this argument from a gene-expression observation into a small-RNA biology study. TRES-2 and TRES-3 carried both higher total tRF abundance and broader detectable repertoires than TRES-1. The shift in internal and 5-prime tRF fractions, together with parent-tRNA and exact-fragment differences, indicates that the tissue states are expressed as distinct RNA product architectures. This is consistent with evidence that tRFs and tiRNAs participate in translational regulation, RNA binding and stress adaptation, and that their functions depend on cleavage position, sequence and cellular context (Yu et al., 2021; Gong et al., 2023; Chen et al., 2024; Kumar et al., 2016; Goodarzi et al., 2015). In gastric cancer, individual fragments already have demonstrated functional relevance (Kohansal et al., 2022; Cui et al., 2022). The present framework adds a systems-level explanation for why particular fragments may emerge: they are produced within a broader tRNA-regulatory state.

The shared tissue-serum sequence catalogue gives this systems view an additional dimension. The 446 exact sequences common to both catalogues identify a concrete molecular territory in which tumour tissue and circulation can be examined together. By focusing on sequence identity, the study provides a rigorous starting point for future paired-sample work: exact tRFs can be tracked across tissue, plasma or serum, evaluated in extracellular particle fractions, and tested with targeted quantitative assays. The current serum literature in gastric cancer already demonstrates the feasibility of absolute tRF measurement in blood (Zhang et al., 2023), while liquid-biopsy research increasingly seeks markers that reflect dynamic tumour biology rather than static disease labels (Han and Lee, 2024; Diaz Del Arco et al., 2024). The present results nominate an organised sequence space for this next step.

The state association with ACRG molecular architecture, RNA-handling programmes and single-cell localisation deepens the biological interpretation. Gastric-cancer taxonomy has repeatedly shown that epithelial, immune and mesenchymal programmes are linked to distinct patterns of progression and treatment response (Cancer Genome Atlas Research Network, 2014; Cristescu et al., 2015; Kumar et al., 2022; Huang et al., 2023; Li et al., 2022; Wang et al., 2021). Our findings place tRNA regulation within this established landscape. The enrichment of MSI in TRES-3 and MSS/EMT in TRES-1 connects the state axis to recognised gastric-cancer biology; the RNA cargo-selection, extracellular-vesicle release and stress-response modules extend it into extracellular-RNA machinery; and the single-cell maps show that the programme is distributed across epithelial, stromal and immune compartments. Together, these observations make TRES a tissue-ecosystem framework rather than a narrow expression signature. Extracellular-RNA biology demands careful molecular definition, and current MISEV guidance provides an important framework for future mechanistic experiments that separate vesicular and non-vesicular routes of RNA carriage (Welsh et al., 2024).

The serum sequencing results supply the clinical-material dimension of the study. The reciprocal loss of the 14-30-nt fraction and gain of the 31-40-nt fraction from CAG to EGC to GC is visually intuitive, statistically ordered and reproducible in leave-one-out summaries. Tissue analyses show how tRNA-regulatory states are associated with direct tRF output, source modules and exact sequences; serum analyses show that circulating small-RNA architecture changes across clinically recognised stages of gastric disease. The combination is especially relevant to early detection, where the challenge is to translate tissue biology into measurements that can be obtained repeatedly and with minimal invasiveness (Han and Lee, 2024; Diaz Del Arco et al., 2024; Janjigian et al., 2021).

The source-tRNA and exact-fragment findings define exploratory candidates. Arg-CCG, Gln-CTG, Leu-CAA, Leu-CAG, Met-CAT and Phe-GAA showed cross-sectional trajectories, and five sequence-coordinate-audited fragments provide a starting panel for validation. Taken together, these results advance a simple but consequential idea: the tRNA system is an organising layer of gastric-cancer biology. It integrates translational capacity, stress adaptation, genomic context, tissue small-RNA output and circulating RNA composition. By showing this organisation across tissue and serum, the study provides a biologically grounded framework for future work on gastric-cancer stratification, RNA-based monitoring and tRNA-directed therapeutic vulnerabilities.

This study is associative and has important limitations. TRES-3 contains a prominent proliferation component, and the available data do not support causal or tRNA-specific mechanistic attribution independent of cell cycle, tumor purity, stromal content, and immune infiltration. The single-cell analysis is descriptive and does not test coordinated cross-lineage regulation. Tissue tRF abundance can be influenced by library depth and compositional dependence despite RPM normalization, deterministic depth-based library selection, and global FDR control. The serum arm includes only three independent participants per group. No perturbation experiment was performed; all molecular, clinical, and mechanistic interpretations are correspondingly qualified.

## 5 Conclusion

This study establishes tRNA regulation as an organizing framework of gastric cancer ecosystem biology, with a circulating small-RNA signature that parallels disease progression from pre-malignant to malignant stages.

## Data Availability

The original serum raw FASTQ files and complete counts/CPM matrix are deposited in GEO under accession GSE343860. Public datasets used in this study were TCGA-STAD, GSE62254 (GPL570; n=300), GSE183904 (40 supplied processed sample matrices), and GSE206785 (24 paired tumour/normal participants; supplementary sensitivity analysis).
